# Frontiers in Pharmacology: Review Manuscript Targeting of the Neutrophil as an Adjunctive Strategy in Non-Small Cell Lung Cancer

**DOI:** 10.3389/fphar.2021.676399

**Published:** 2021-06-08

**Authors:** Ronald Anderson, Ada Gabriela Blidner, Bernardo Leon Rapoport

**Affiliations:** ^1^ Department of Immunology, Faculty of Health Sciences, University of Pretoria, Pretoria, South Africa; ^2^ Laboratory of Immunopathology, Institute of Biology and Experimental Medicine CONICET, Buenos Aires, Argentina; ^3^ The Medical Oncology Centre of Rosebank, Johannesburg, South Africa

**Keywords:** adjunctive therapy, immunosuppression, lung cancer, myeloid-derived suppressor cells, neutrophils, non-small cell lung cancer, tumor-associated neutrophils

## Abstract

Lung cancer remains the leading cause of cancer mortality in the United States, with non-small cell lung cancer (NSCLC) accounting for around 85% of cases. Of particular concern is the poor responsiveness of this malignancy to therapy, resulting in a very low 5-year survival rate (17.4%) and a prominent tendency to progress to metastatic disease. A number of very recent studies, both pre-clinical and clinical, have implicated the neutrophil in both the pathogenesis and unsatisfactory response to therapy of NSCLC. In this context, movement of neutrophils into the tumor microenvironment (TME) is a common feature of NSCLC. Indeed neutrophils are the dominant type of immune cell in the NSCLC TME, creating a highly immunosuppressive milieu that is not only conducive to tumor growth and spread, but also represents a significant obstacle to the success of anti-tumor therapy, especially novel immunotherapies. The clinically relevant adverse impact of a neutrophil predominance both systemically and in the TME of patients with NSCLC is underscored by the negative prognostic value of both a persistent neutrophilia and, in particular, a high (≥5) neutrophil:lymphocyte ratio. On a more positive note, however, recognition of the involvement of the neutrophil in both the pathophysiology of NSCLC and treatment failure has enabled identification of neutrophil-targeted strategies that have the potential to serve as adjuncts to standard anti-cancer therapies, including immunotherapy. These strategies together with a consideration of the immunosuppressive, pro-tumorigenic properties of the neutrophil represent the major thrusts of this review.

## Introduction

Lung cancer is usually diagnosed at a late stage and remains the leading cause of death from cancer among men and women, accounting for approximately 25% of the 9.6 million total cancer deaths recorded globally in 2018 with an overall five-year survival rate of 5% in the case of advanced, metastatic disease (Didowska et al., 2016). Overall, 85–90% cases of lung cancer are non-small cell lung cancers (NSCLCs), the most common being adenocarcinoma (40%), squamous cell carcinoma (25–30%) and large cell carcinoma (10–15%) (Grapatsas et al., 2017). Although recent advances in treatment have demonstrated that NSCLC responds to immunotherapy, a large number of patients remains resistant to anti-cancer therapy (Horvath et al., 2020). One of the main barriers to advancing this field is the lack of validated, predictive biomarkers of response to treatment in patients with this disease. Validated biomarkers identified to date that are predictive of responsiveness to immunotherapy in NSCLC include the tumor mutational burden (TMB) and expression of programmed cell death ligand (PD-L1) (Pharaon et al., 2020). Clearly, however, there is a need to identify additional biomarkers that will enable clinicians to accurately identify patients who will benefit from this treatment modality. In this context, immunosuppressive neutrophils, including myeloid-derived suppressor cells (MDSCs), contribute significantly to the inadequate response to targeting immune checkpoint inhibitors (ICIs) in patients with NSCLC. Currently, the neutrophil:lymphocyte ratio (NLR) is the only neutrophil-related biomarker utilized by clinicians in the management of patients with NSCLC (Rapoport et al., 2020). However, the NLR is a prognostic biomarker. To date, neutrophil-related predictive biomarkers have not been identified.

The current review is focused primarily on the remarkable propensity of NSCLC to recruit neutrophils to the tumor microenvironment (TME) and to harness the immunosuppressive potential of these cells to promote tumor growth and invasion. Key topics covered include: 1) an overview of evidence linking neutrophils to the pathogenesis of NSCLC; 2) mechanisms utilized by NSCLC to co-opt neutrophils; 3) similarities and differences between neutrophils and other types of immune suppressor cells in the TME, as well as interactions between these cells; 4) mechanisms utilized by neutrophils both to suppress the anti-tumor reactivity of tumor-infiltrating lymphocytes (TILs) and to exclude these cells from the TME; and 5) the involvement of neutrophils and sub-types of neutrophil-derived suppressor cells in angiogenesis and tumor invasion. Finally and importantly, the review will conclude with an overview of adjunctive neutrophil-targeted pharmacological- and monoclonal antibody-based strategies that have the potential to complement various types of standard anti-cancer therapy, most prominently immunotherapy. These anti-inflammatory/immunosuppressive mechanisms are in many cases not specific to neutrophils, but are rather multifaceted, affecting various types of immune and inflammatory cells.

## Partnering of Non-Small Cell Lung Cancer With Neutrophils as a Strategy to Promote Tumorigenesis

Much of the evidence implicating neutrophils in the pathogenesis of both progression of NSCLC and attenuation of treatment efficacy, has been derived from routine hematological investigations. These include enumeration of the number of circulating neutrophils and lymphocytes (Schernberg et al., 2018; Rapoport et al., 2020) and using this data to calculate the NLR by dividing the total number of neutrophils by the total lymphocyte count. Although awaiting validation, a resultant NLR most commonly of ≥5 (although some studies use lower cut-off values of 3 or 4) is indicative of subclinical systemic inflammation and immunosuppression. Measured prior to and during anti-cancer therapy, this, in turn, has been reported in numerous studies (too numerous to be listed here), to be associated with a negative clinical outcome and poor responsiveness to various types of anti-cancer treatments. These include chemotherapy, radiotherapy and surgery (Sarraf et al., 2009; Tomita al., 2011; Peng et al., 2015; Scilla et al., 2017; Mizuguchi et al., 2018; Doi et al., 2019), and, more recently, inhibitory immune checkpoint-targeted therapies (Banna et al., 2020; Huang and Shen, 2020; Jin et al., 2020; Rapoport et al., 2020b). Although of particular relevance to NSCLC, the prognostic value of the NLR has also been described in many other common malignancies (Donskov, 2013; Howard et al., 2019). As alluded to later, measurement of the platelet:lymphocyte, as well as the monocyte:lymphocyte, ratio may also have prognostic potential.

### Analysis of the Cellularity of the Non-Small Cell Lung Cancer Tumor Microenvironment in the Clinical Setting

Few studies, on the other hand, have focused on the presence and immunosuppressive/adverse prognostic activities of neutrophils within the tumor TME of NSCLC. In this context, an earlier study undertaken by Gentles et al. (2015) *.* based on “CIBERSORT” genomic/transcriptomic profiling to estimate the immune cell composition of tumor biopsies derived from the gene expression signatures of approximately 18,000 human tumors, encompassing 39 different types of malignancy, is noteworthy (Gentles et al., 2015). This approach resulted in the identification of 22 different types of tumor-associated leukocytes in the TMEs, the presence of which was correlated with overall survival (OS) (Gentles et al., 2015). In this context, the authors identified significant, inverse prognostic associations between gene expression profiles typical of polymorphonuclear leukocytes (neutrophils) that were evident “across the cancer landscape”, most prominently with the lung adenocarcinoma variant of NSCLC, as well as with breast cancer, although subtypes of the latter malignancy were not mentioned (Gentles et al., 2015).

More recently, (Kargl et al., 2017). investigated the immune cell composition of the NSCLC TME more definitively (Kargl et al., 2017). These authors used a 21-marker flow cytometry panel with the capability of detecting 51 different types of immune cells in single cell suspensions prepared from lung cancer tissue and non-adjacent normal lung tissue (Kargl et al., 2017). This procedure was combined with detection of tumor-reactive TILs using T cell receptor *β*-chain rearrangements and functional assays based on increased numbers of clonally expanded T cells reactive with tumor-specific antigens (TSAs) (Kargl et al., 2017). In an initial series of experiments using biopsy material from 10 patients with NSCLC (adenocarcinoma), the authors observed that T cell clonality in the NSCLC TME was common, although expansion was variable and no association of smoking (drives tumor mutational load) with clonality was evident (Kargl et al., 2017). Importantly, neutrophils were found to “dominate the immune landscape of NSCLC”.

These authors then profiled the cellularity and function of immune cells isolated from the tumor tissue and non-adjacent normal tissue of 73 patients with NSCLC who were undergoing curative-intent surgical resection (Kargl et al., 2017). They observed that CD45^+^ immune cells predominated in tumor tissue, accounting for 75% of tumor cellularity that was confirmed by immunohistochemistry and was 3-fold higher than that of non-tumor tissue. Numbers of B-lymphocytes were 7-fold higher in NSCLC tumor tissue relative to normal tissue. Numbers of regulatory T cells (Tregs) were increased and Th1 cells decreased, correlating positively and negatively with tumor size, respectively (Kargl et al., 2017). Although CD8^+^ T cells in tumor tissue expressed the activation marker CD69, they also co-expressed the ICIs, programmed cell death protein 1 (PD-1) and T cell immunoglobulin and mucin domain-containing protein 3 (TIM3), while multiple cell types expressed PD-L1, including macrophages, monocytes and neutrophils (Kargl et al., 2017).

Cells of the myeloid lineage accounted for 50% of CD45^+^ immune cells in NSCLC tissue. Neutrophils were most abundant at approximately 20%. Although the numbers of these cells were equivalent in both tumor and non-tumor tissue, the former were mostly located in the tumor stroma (Kargl et al., 2017). Importantly, strong negative correlations were detected between the numbers of neutrophils and those of CD4^+^CD8^+^ cells in the TMEs of both subtypes of NSCLC, but not in non-adjacent lung tissue, while PD-1-expressing CD4^+^ T cells correlated with tumor size and clinical stage (Kargl et al., 2017).

In a follow-up study published almost two years later, (Kargl et al., 2017) investigated associations of neutrophil colonization of the TME in patients with NSCLC with anti-tumor immunity according to intratumoral migration of CD8^+^ T cells, as well as with responsiveness to PD-1-/PD-L1 monoclonal antibody-based targeting. Three cohorts of patients with NSCLC were recruited, namely those mentioned above (*n* = 73) receiving lung resection with curative intent and two groups who had either received (*n* = 28) or were receiving immunotherapy (*n* = 52). Cellularity of the TME was determined by sensitive analysis of single cell suspensions using the combination of flow cytometry, gene expression and multiplexed immunohistochemical analyses (Kargl et al., 2019). Briefly, the authors reported that the TME of NSCLC could be identified as one of two different types, these being “Active” or “Myeloid”, according to the presence of low or high numbers of neutrophils within the tumor stroma, respectively (Kargl et al., 2019). The “Myeloid” type referred specifically to colonization of the TME by neutrophils, but not macrophages, monocytes or myeloid-derived suppressor cells (MDSCs) and was associated with depletion of CD4^+^ and CD8^+^ T cells. Based on calculation of the ratio of CD8^+^ T cells within the tumor mass and neutrophils in the stroma, the authors were able to calculate a CD8^+^ cell/neutrophil (CD66 b^+^) ratio, with lower ratios being predictive of poor responsiveness to immunotherapy (Kargl et al., 2019).

More recently, Mitchell et al. (2020), using a range of cellular/immunological and molecular technologies investigated the association between systemic and intratumoral neutrophil expansion in a cohort (*n* = 66) of NSCLC patients with predominantly lung adenocarcinoma (71%), 46% of whom had stage 1 disease. Pre-operatively, circulating neutrophil counts were found to correlate with increased tumor burden, as well as with increased levels of the neutrophil-mobilizing cytokines, interleukin (IL)-1β, IL-17A and tumor necrosis factor-α (TNF-α).

Analysis of resected tumor tissue revealed that the intratumoral neutrophil burden was more strongly associated with decreased cytotoxic T-cell trafficking than with other prominent indices of the immune milieu of the TME, including expression of PD-L1, the TMB and histology. The intratumoral neutrophil burden was also associated with impairment of both anti-tumor T cell cytotoxicity and expression of genes involved in interferon-γ (IFN-γ) signaling (Mitchell et al., 2020).

These authors concluded that neutrophil expansion, both systemically and in the TME of patients with NSCLC “reflects protumorigenesis and immunosuppressive processes that manifest as worse OS in patients undergoing NSCLC resection” (Mitchell et al., 2020).

### Preclinal studies Linking Intratumoral Neutrophils to Tumorigenesis and Failure of Immunotherapy

Although investigations in the clinical setting of NSCLC are few, the aforementioned findings reported by Kargl et al. (2019) and Mitchell et al. (2020) are, however, supported, by a number, albeit small, of preclinical studies of experimental tumorigenesis. One such study involving mice bearing genetically engineered Kras G^12D^-/IL-17A-expressing lung tumors, clearly demonstrated dependence of both tumor progression and resistance to PD-1 targeted immunotherapy on neutrophil recruitment that was driven by tumor-derived IL–17A (Akbay et al., 2017). In another experimental study that was an extension of that reported by Kargl et al. (2019) described above, the authors demonstrated the potential of SX-682, a potent allosteric inhibitor of the neutrophil-mobilizing CXC chemokine receptors, CXCR1/2, to overcome neutrophil-associated attenuation of the efficacy of PD-1-targeted immunotherapy in a murine model of lung squamous cell carcinoma, a malignancy that is also associated with significant neutrophil infiltration.

Although promising, currently available evidence linking tumor progression and treatment failure to recruitment of neutrophils to the TME of patients with NSCLC, is nevertheless somewhat limited, necessitating additional stringently controlled studies, particularly in the clinical setting, to enable firm conclusions. This contention is supported by the findings of another recent study that reported somewhat different findings to those mentioned above (Tamminga et al., 2020). These authors used CIBERSORT technology to analyse the immune cell infiltrates of tumor tissue from NSCLC patients (*n* = 1,022; 71.5% with adenocarcinoma; 76% smokers), the majority of whom had early stage disease (stages I and II; *n* = 922). Immune cell infiltrates were then correlated with OS. For the group of adenocarcinoma patients, improved OS was significantly associated with resting mast cells, memory B cells and resting CD4^+^ T cells [respective hazard ratios (HRs) of 0.96 (*p =* 0.01), 0.97 (*p* = 0.01) and 0.98 (*p =* 0.04)]. On the other hand, the percentages of neutrophils, T follicular helper (Tfh) cells and M0 and M2 macrophages were associated with significantly decreased OS rates [respective HRs of 1.08 (*p* < 0.01); 1.07 (*p =* 0.01); 1.02 (*p* = 0.01) and HR = 1.02 (*p* = 0.03)] (Tamminga et al., 2020). Not surprisingly, these findings are consistent with the co-existence of augmentative suppressive interactions between different types of suppressor cells in the TME of NSCLC (Wang et al., 2018; Ferreira et al., 2020).

## Neutrophil Subsets and Characterization

Considering the plasticity of myeloid populations, the dynamic changes in surface markers and the wide variety of phenotypes, these heterogeneous populations embody the concept that untangling the distinction between neutrophils and myeloid-derived suppressor cells (MDSCs) remains a complicated task. Neutrophils and MDSCs share a common progenitor originating in the bone marrow, which, in physiological conditions, gives rise to neutrophils, macrophages and dendritic cells. During pathological settings (i.e., sepsis, trauma, infection, cancer) myeloid progenitors pause maturation and become tolerogenic MDSCs (Gabrilovich and Nagaraj, 2009). In mice and humans, two main MDSC subpopulations have been described, polymorphonuclear (PMN-) and monocytic (M-) MDSCs. Morphologically they are both very similar to their effector mature counterparts, neutrophils and macrophages respectively; however, they differ in surface marker expression and functionality (Bronte et al., 2016). In order to discriminate human PMN-MDSCs from neutrophils (PMNs), gradient centrifugation (1.077 g/ml), as well as detection of higher LOX-1 expression in PMN-MDSCs, are presently used as valid technics (Zhou et al., 2018; Si et al., 2019). However, recent evidence suggests that different PMN populations coexist in tumor bearing mice and cancer patients. In this context, three different PMN populations were found infiltrating the tumor: 30% corresponded to neutrophil-like PMNs, 16% classical PMN-MDSCs and 55% were activated PMN-MDSCs (Veglia et al., 2021).

Genetically, the three sub-populations show distinct gene expression profiles, ranging from non-suppressive PMNs, to immature/suppressive PMN-MDSCs (both found mainly in the spleen of healthy or tumor-bearing mice), and high chemokine/ER stress responses in activated PMN-MDSCs, residing mainly in tumors (Veglia et al., 2021). Interestingly, CD14 was shown to be a good marker of activated PMN-MDSCs in tumor-bearing mice, which was also associated with poor prognosis in cancer patients (Veglia et al., 2021). Supporting these findings, Zilionis et al. (2019) described similar neutrophil populations. They compared healthy and tumor bearing lungs in NSCLC patients and experimental models in mice. Interestingly they found 6 distinct neutrophil phenotypes and, concordantly with Veglia and collaborators, they found that classical PMN/neutrophils were enriched in blood and healthy lungs, while subtypes enriched or exclusive to tumor-bearing lungs depicted an increased expression of chemokine receptors, pro-angiogenic factors and ER stress related genes (*XBP1*, *SPP1* and *CSF1* were found to be shared by tumor-specific PMNs from both articles). Additionally, both articles described intermediate PMN phenotypes that originate from classical neutrophils, but do not directly convert into activated PMN-MDSCs/pro-tumoral TANs, further demonstrating the intricate landscape of neutrophil populations in cancer settings.

Notably, the ER stress response was also associated with pro-metastatic PMN populations, while activation of the XBP1/IRE1 pathway resulted in an increase of LOX1 gene expression, the signature of suppressive PMN-MDSCs (Veglia et al., 2021).

To add additional layers of complexity, anti-tumor neutrophils can be reprogrammed in the TME and become tolerogenic. Such as occurs with Th1/Th2 or M1/M2 phenotypes, neutrophils were described as IFN-β-induced pro-inflammatory N1 and TGF-β stimulated anti-inflammatory N2. Since no differential markers between N1 and N2 phenotypes were described, these populations probably indicate different functional states, due to neutrophil plasticity, depending on the soluble mediators present in the tumor tissue (Fridlender et al., 2009; Shaul et al., 2016). Moreover, N1 tumor-associated neutrophils (TANs) present hypersegmented nuclei compared to N2 TANs, suggesting that these cellular compartments could be different stages in intratumor neutrophil maturation. Additionally, recent findings show that activation of ER stress signaling enhances the PMN-MDSC marker, LOX-1, in TANs, further supporting the notion that TANs are a heterogenous population of polymorphonuclear cells in different stages of maturation and polarization (Wu and Zhang, 2020; Veglia et al., 2021).

In lung cancer patients, accumulation of the low-density fraction (LDF) blood CD66 b^+^ PMNs correlates not only with poor prognosis, but also with disease burden, ranging from a median of 0.1% of total LDF PBMCs in early stages to 7.5% in advanced stages. This effect was also accompanied by an increase in the NLR (Shaul et al., 2020). CyTOF analysis depicted three neutrophil populations based on CD10 marker expression, previously shown to separate mature CD66b^+^ immune-stimulatory from CD10-negative immature neutrophils (Marini et al., 2017). CD10^low/neg^ LDM neutrophils express high levels of PD-L1, suggesting they could be targeted by PD-L1 neutralizing therapies. In line with previous findings, the authors described the transition of high-density fraction (HDF) neutrophils to LDF neutrophils, further stimulated by TGF-β signaling. The manuscript also described the reverse neutrophil transition from LDF to HDF. While the first event might represent a change in density due to degranulation, supported by equal expression of CD10, the second, might suggest a conversion of immature neutrophils to more mature CD10^+^ HDF cells (Shaul et al., 2020). In this sense, although different authors refer to LDF PMN cells as N2 TANs or PMN-MDSCs, there are phenotypic consistencies indicating that LDF PMNs, are more immature and more immunosuppressive and represent a tumor-promoting cell compartment with poor prognosis in lung cancer patients.

## Neutrophil Recruitment and Function in Lung Cancer

The physiology of the lung, the continuous exchange with the external medium and the extensive vascular irrigation, are crucial factors influencing infiltration of cells of innate and adaptive immunity into to the organ. Neutrophils, one of the main immune populations present in the lung, have distinctive roles during homeostasis, inflammation and cancer.

### Mechanisms of Tumor-Associated Neutrophil Recruitment

Accumulation of systemic and intratumoral neutrophils has been associated with higher tumor progression in experimental models (Schernberg et al., 2018; Pfirschke et al., 2020; Rapoport et al., 2020) and poor prognosis in patients (Kargl et al., 2019). Concordantly, soluble factors that increase bone marrow exit and tumor recruitment of PMN cells correlate with therapy resistance and tumor malignancy. Interleukin-8 (IL-8) also known as C-X-C motif chemokine ligand 8 (CXCL8), shows pleiotropic roles in cancer settings, including promotion of angiogenesis, epithelial-mesenchymal transition (EMT), immunosuppression and metastasis. Produced by monocytes, endothelial cells and epithelial cells, its action is mediated by CXCR1 and CXCR2. Immunologically, IL-8 enhances chemotaxis of neutrophils to the tumor site and its levels correlate with disease burden, advanced tumor stage and poor prognosis (Waugh et al., 2008). Association of IL-8 with ICI therapy failure was observed in NSCLC, as well as in melanoma and renal cell carcinoma (RCC) (Sanmamed et al., 2017; Schalper et al., 2020). In a retrospective analysis, an early increase in serum levels of IL-8 predicted poor outcome in advanced NSCLC and melanoma patients treated with ICI-targeted monoclonal antibodies (mAbs) (Sanmamed et al., 2017). Moreover, baseline elevated levels of IL-8 (cut-off: 23 pg/ml) were associated with adverse outcomes in squamous NSCLC, non-squamous NSCLC, melanoma and renal cell carcinoma (RCC) and strongly correlated with circulating neutrophils not only in the ICI-treated arms, but also in docetaxel-non-squamous NSCLC and single-agent everolimus-treated RCC patients (Schalper et al., 2020).

In addition, IL-8 levels correlated with less expression of the antigen presenting machinery in myeloid cells, a hallmark of immunosuppressive activity; whether this is due to increased emergency myelopoiesis and accumulation of immature cells or to the effect of the TME, remains to be determined (Bakouny and Choueiri, 2020). Additionally, the neutrophil receptor, CXCR2 binds to the chemokines, CXCL3, CXCL5 and CXCL7, which are increased in *STK11/LKB1* deficient mice models and human lung cancer cell lines and mediate, along with IL-6 and IL-1α, immunosuppressive neutrophil recruitment to the tumor, spleen and peripheral blood (Koyama et al., 2016). Moreover, CXCL1 (also known as Gro-1) expressed by mouse and human lung cancer cell lines, fosters TAN recruitment to the tumor. Silencing of CXCL1 in a 3LL mouse lung cancer model inhibits tumor growth and TAN mediated immunosuppression (Yuan et al., 2016). In lung cancer patients, CXCL1^high^ expressing tumors showed reduced progression-free survival (PFS) and was proposed as a biomarker of disease progression (Cao et al., 2017). Finally, immunosuppressive neutrophil recruitment to lung tumors can be remotely stimulated by osteoblasts via a CXCR2/sRAGE (soluble receptor for advanced glycation end-products)-dependent mechanism (Engblom et al., 2017).

### Mechanisms of Activity of Tumor-Associated Neutrophils

As stated before, N1 and N2 TANs constitute opposite forces in relevant tumor promoting processes as immunosuppression, angiogenesis and metastasis. Although these seem separate cancer manifestations, they share common molecular mechanisms that generate a positive feedback loop that ends in cancer progression. To start with classical neutrophil functions, regulation of tumor specific immune responses is one of the hallmarks of TANs. Antigen presentation, release of soluble factors, enzymatic activity and reactive oxygen species (ROS) can either foster or impair anti-tumor immunity. During the early stages of tumor evolution, according to the immunoediting theory, tumor cells recruit inflammatory innate and adaptive immune cells that destroy sensitive clones, while selecting resistant ones (Dunn et al., 2002). In this phase, N1 TANs exert cytolytic functions primarily through ROS production and stimulate tumor specific T cells by antigen presentation and pro-inflammatory cytokine production such as IL-1β, IL-6, TNF-α, or IL-17A. In this context, freshly isolated TANs from early stage lung cancer patients were unable to suppress polyclonally activated T cells *in vitro* (Eruslanov, 2017). As tumor progression continues, resistant clones produce high levels of neutrophil recruiting chemokines and immunosuppressive cytokines that transform the anti-tumor immune response, which is replaced by immunoregulatory innate immune cells and dysfunctional T cells. Consistently, N1 TANs shift to immature-like myeloid suppressor cells, as discussed above, namely PMN-MDSCs or N2 TANs. Immunosuppressive strategies involve arginase-1 enzymatic activity, which depletes arginine from the extracellular milieu, leaving proliferating T cell clones stripped of an essential amino acid necessary for antigen recognition and survival (Gabrilovich and Nagaraj, 2009). Additionally, ROS and nitric oxide (NO) production impair intact T cell receptor function and also induce apoptosis in T cells (Schmielau and Finn, 2001). Among regulatory cytokines, IL-10 and TGF-β1 are produced by N2 TANs and impair T cell mediated cytotoxicity.

Interestingly, immunosuppression is not the only tumor-promoting activity of this cell population, and several molecules secreted by neutrophils share pro-angiogenic and pro-metastatic potential. Matrix metalloproteases (MMPs) degrade extracellular matrix and promote tumor extravasation and metastatic niche formation (Quintero-Fabian et al., 2019); however, these molecules also stimulate vascular endothelial growth factor (VEGF) release and vascular remodeling (Deryugina et al., 2014). Moreover, neutrophils can directly secrete VEGF and induce angiogenesis (Mantovani et al., 2011). In addition, G-CSF has been linked to Bv8 upregulation and neutrophil-induced angiogenesis (Shojaei et al., 2007), while elastase and cathepsin G are two neutrophil-derived proteinases that induce tumor growth, metastasis and immunosuppression (Moroy et al., 2012; Oberg et al., 2019).

Metastatic potential of PMNs has been widely documented; in fact, not only soluble factors produced by these cells are responsible for metastasis promotion. The ability of neutrophils to travel through the bloodstream and colonize distant organs endows them with the capacity to chaperone circulating tumor cells to enhance tissue colonization. Although PMN-tumor cell interaction is still an ongoing hypothesis, evidence shows that ICAM-1 expressed on tumor cells binds to Mac-1 (Integrin αΜ + Integrin β2) expressed on PMNs (Mollinedo, 2019). Moreover, tumor cells and PMNs were shown to form heterotypic clusters able to migrate across the endothelium (Mollinedo, 2019). Concerted degranulation and neutrophil extracellular trap (NET) formation are additionally involved in metastasis. In NSCLC, the role of PMNs in poor prognosis and metastatic status is compelling. As stated before in this manuscript, the NLR ratio is becoming a reliable biomarker for prognostic and predictive stratification in NSCLC (Boscolo et al., 2020). Moreover, PMNs are recruited to distant organs through CXCR2/4 signaling and foster pre-metastatic niche formation (Wu, 2020).

**FIGURE 1:**
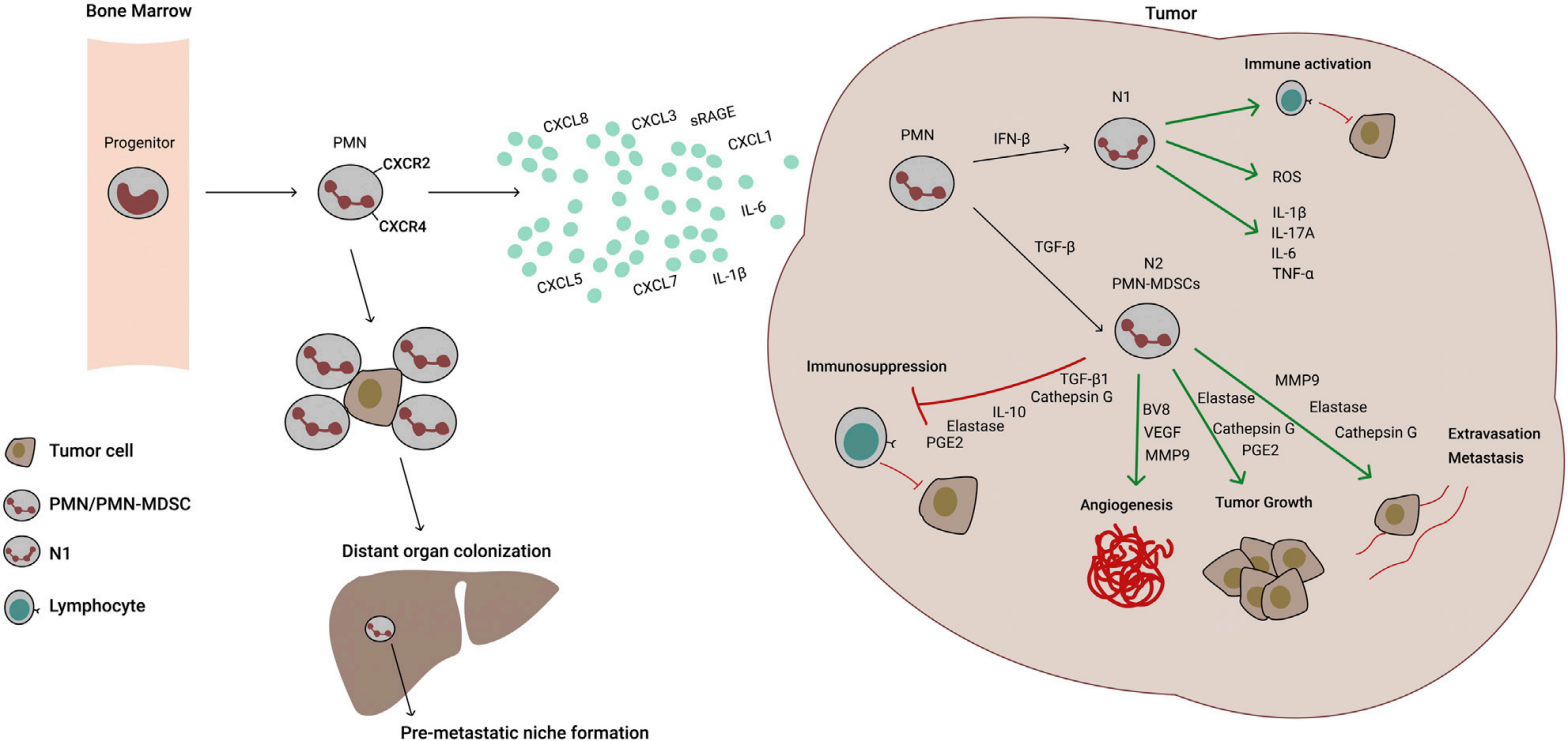
Molecular mechanisms of neutrophil recruitment and activity in lung cancer. Polymorphonuclear (PMN) progenitors mature to PMN cells and exit the bone marrow through CXCR2 sensing of tumor secreted chemokines and cytokines (IL-8/CXCL8, CXCL1, -3, -5, -7, IL-6 and IL-1β). Once in the tumor, PMNs can differentiate into N1 or N2 tumor‐associated neutrophils (TANs). N1 TANs are pro-inflammatory, have high expression of the antigen-presenting machinery and Reactive Oxygen Species (ROS) production, but low arginase-1 activity. Through antigen presentation and inflammatory cytokine secretion, they stimulate anti-tumor T cells and exert direct killing of tumor cells mediated by ROS and nitric oxide (NO) production. Under TGF-β induction, however, PMN differentiate into N2 TANs or PMN-MDSCs, which are less mature, immunosuppressive and foster angiogenesis, tumor growth and metastasis. In contrast to N1 TANs, N2 TANs cells downregulate antigen presentation, but express high levels of PD-L1, arginase-1, ROS and NO. Additionally, they produce crucial soluble mediators of neovascularization, tumor cell growth, extravasation and metastasis. Additionally, CXCR2/4 signaling recruits PMNs to seeding organs promoting pre-metastatic niche formation. PMNs are able to protect CTCs (circulating tumor cells) and escort them to distant organs, improving metastatic process.

The aforementioned events are summarized in Figure 1.

In an effort to elucidate PMN effects on tumorigenesis and metastatic processes, new experimental strategies explore the simultaneous study of tumor cells and inflammatory cells. An example of these comprehensive approaches are 3D organoids that recapitulate essential components of the organ´s structure and functions. Although lung organoids have been established, there are still no studies encompassing carcinogenesis process and PMN biology. However, a study by Sachs et al. showed that PMNs depict an enhanced recruitment to inflamed lung organoids, compared to mock controls, via CXCL10-and RANTES -mediated engagement (Sachs et al., 2019), which are two chemokines present in NSCLC. Patient -derived xenografts (PDXs) additionally enable the study of innate responses on diverse cancer treatments. These partially immunosuppressed mice, NOD-SCID gamma (NSG), lack an adaptive immune system, but retain functional innate immune responses. A work by Martin Ruiz et al. (2020), depicted a novel effect of PD-1 blockade on PMNs; anti-PD-1 antibody binds to the cell surface of PMNs both by recognizing the antigen PD-1 and by (Fc)-gamma receptors (FcγR) attachment. In turn, PMNs become activated and foster tumor necrosis by NO and ROS production (Martin-Ruiz et al., 2020).

## Targeting Neutrophils as an Adjunctive Therapeutic Strategy in Non-Small Cell Lung Cancer

Avoiding the potential threat of severe infective complications in the setting of an already serious disease clearly poses a challenge to the development of effective neutrophil-targeted adjunctive therapies in NSCLC. Nevertheless, several innovative, precision targeted approaches, some preclinical, that avoid the risks of neutrophil depletion and abrogation of key host defense mechanisms are currently in the pipeline. Several of those, that we consider most promising, are summarized as follows and are dealt with in greater detail in the final section of this review:

• Disabling MDSCs in particular, as well as other types of suppressor cells that drive their development such as Tregs and M2-type macrophages;
• Neutralizing tumor-derived chemokines that promote the influx of neutrophils and MDSCs into the TME, while sparing other chemoattractants and their receptors that are at the forefront of anti-infective host defense such as C3a, C5a, leukotriene B4 and bacterial formyl peptides;
• Inactivating neutrophil-modulating cytokines, specifically IL-1β and IL-17A;
• Although largely unexplored, modulation of the neutrophil-targeted pro-inflammatory activities of platelets also represents a novel strategy in the adjunctive immunomodulatory therapy of NSCLC and possibly other types of advanced malignancies;
• Perhaps somewhat surprisingly, transplantation of fecal microbiota in the clinical setting of advanced malignancy represents another potential strategy to attenuate the numbers of cells with a MDSC-like phenotype.

It should be noted that several extensive recent reviews have focused on pharmacological and biological strategies that have the potential to target neutrophils, including in the setting of various types of cancer (Granot, 2019; Poeta et al., 2019; Shaul and Fridlender, 2019; Vilgelm and Richmond, 2019; Villanueva, 2019; Wu et al., 2019; Rapoport et al., 2020a). As mentioned above, however, only those that are most recent and seemingly innovative and promising in the setting of NSCLC, in our opinion, are covered in detail here. In addition to ROS, NO, TGF-β1 and IL-10, other putative neutrophil/MDSC-derived immunosuppressive mechanisms that have been reviewed elsewhere include: 1) depriving T cells in the TME of arginine, tryptophan and cysteine via release of arginase1, indoleamine 2,3-dioxygenase and sequestration of cystine, respectively (Srivastava et al., 2010; Yu et al., 2013b; Treffers et al., 2016; Groth et al., 2019); and 2) release of adenosine that promotes intracellular production of immunosuppressive 3′,5′-cyclic adenosine monophosphate (Groth et al., 2019; Rapoport et al., 2020).

## Strategies That Target Myeloid-Derived Suppressor Cells

Several cytokines, seemingly present at high concentrations in the TME, specifically granulocyte-macrophage colony stimulating factor (GM-CSF) and TGF-β1, derived from tumors, as well as resident structural and infiltrating immune/inflammatory, cells have been linked to the transition of neutrophils in the TME to a MDSC-like phenotype in various malignancies (Hong, 2016; Jayaraman et al., 2018).

### Targeting Granulocyte-Macrophage Colony Stimulating Factor

In the case of GM-CSF, exposure of neutrophils to this growth factor has recently been reported to selectively induce overexpression of fatty acid transport protein 2 (FATP2) that facilitates cellular uptake of long- and very long-chain fatty acids (Veglia et al., 2019). GM-CSF-mediated upregulated expression of FATP2 was linked to triggering of the signal and activator of transcription (STAT5), and, importantly resulted in reprogramming of neutrophils to a MDSC-like phenotype (Veglia et al., 2019). Acquisition of immunosuppressive activity was associated with increased uptake of arachidonic acid by these neutrophil-derived MDSCs, resulting in excessive production of immunosuppressive prostaglandin E_2_ (PGE_2_) and disease progression (Veglia et al., 2019). In murine models of experimental tumorigenesis (specifically lymphoma, cancers of the lung and colon), disease progression was significantly attenuated by treatment of immunocompetent mice, but not immunodeficient mice, with a pharmacological inhibitor of FATP2. This agent known as lipofermata {5′-bromo-5-phenyl-spiro [3H-indole-3,2′ (3′H)-[1,3,4] thiadazol]-2(1H)-one} was administered subcutaneously at a dose of 2 mg/kg twice daily administered 8–10 days after initiation of tumorigenesis and tumor growth monitored for up to 19 days depending on tumor type. Protection was most effective, however, when lipofermata was co-administered with ICI (PD-1 or CTLA4)-targeted mAbs. In addition, targeted deletion of the gene encoding murine neutrophil FATP2 also abrogated the transition of these cells to MDSCs following exposure to either GM-CSF or arachidonic acid (Veglia et al., 2019).

In an even more recent and somewhat similar preclinical study, Adeshakin et al. (2020) confirmed that the pro-tumorigenic activity of TME-derived GM-CSF was indeed achieved by upregulation of FATP2 on neutrophils with resultant transition of these cells to a MDSC phenotype. There were, however, several notable differences between the two studies. In their study, Adeshakin et al. (2020) reported that the transcription factor, STAT3, as opposed to STAT5, was mechanistically involved in GM-CSF-mediated upregulated expression of FATP2 on MDSCs. Secondly, that MDSC-mediated immunosuppression resulted primarily from production of ROS by a mechanism related to lipid-induced mitochondrial oxidative stress that presumably complements PGE_2_-mediated immunosuppression (Veglia et al., 2019; Adeshakin et al., 2020).

As with Veglia et al. (2019), Adeshakin et al. (2020) also observed that tumor progression in murine models of experimental cancer therapy, following subcutaneous administration of lung cancer and melanoma cell lines (LLC and B16F10, respectively), was significantly attenuated by treatment with lipofermata. In addition, the combination of lipofermata with a PD-L1-targeted mAb resulted in augmentative inhibition of tumor progression (Adeshakin et al., 2020).

Targeting of MDSCs via antagonism of GM-CSF and FATP2 is clearly a plausible and possibly effective adjunctive therapeutic strategy in the clinical setting of NSCLC and other types of malignancy. Currently, however, it remains impracticable due to the key role played by GM-CSF in host defense and the uncertain toxicity profile of lipofermata, respectively. Pending the advent of innovative strategies to overcome these challenges, targeting of key MDSC-derived mediators of immunosuppression, such as PGE_2_ and ROS, with inhibitors of cyclooxygenases and NADPH oxidase (NOX) isoforms, respectively, remain promising options. Interestingly, the anti-diabetic agent metformin (dimethylbiguanide) also represents an alternative strategy to target FATP2. In this context, metformin has been reported to inhibit STAT3 (Xu et al., 2019), which, as described by Adeshakin et al. (2020), appears to be a key event in promoting upregulated expression of FATP2. Moreover, it is noteworthy that diabetic patients treated with metformin appear to have a lower propensity for cancer development (Saraei et al., 2019).

### Targeting TGF-β1

Although immunosuppressive granulocytic MDSCs and N2 neutrophils appear to share a common identity, this issue remains unresolved (Ostrand-Rosenberg and Fenselau, 2018). Nevertheless, TGF-β1 produced by tumor cells, Tregs and stromal cells in the TME plays a key role in the transition of infiltrating neutrophils to an immunosuppressive phenotype (Fridlender et al., 2009; Batlle and Massagué, 2019; Masucci et al., 2019). Targeting of TGF-β1 therefore represents a potential strategy in the adjunctive therapy of NSCLC and other types of advanced malignancy (Huynh et al., 2019). This strategy is attainable via the engineering of mAbs that directly neutralize TGF-β1 or its receptors (Ciardiello et al., 2020). In addition, small molecule inhibitors of TGF-β1 receptor signaling, such as galunisertib, an inhibitor of TGF-β1 receptor 1 kinase, represents an alternative strategy (Huynh et al., 2019; Liu et al., 2021). However, given the pleiotropic functions of TGF-β1, including its role in immune homeostasis, direct targeting of this cytokine or its receptor may pose serious safety considerations. In this context, it is noteworthy that galunisertib was withdrawn on January 30, 2020 by its manufacturer (Eli Lilly and Company) due to cardiotoxicity. Currently, two small clinical trials focused on the therapeutic potential of novel TGF-ß inhibitors are ongoing. The first of these is a phase I/II trial (*n* = 60 partipants needed) involving fresolimumab, a mAb that targets all three isoforms of TGF-β, combined with radiotherapy in patients with early stage NSCLC (NCT02581787 scheduled for completion in December 2021) (Huang et al., 2021).The second, is a phase Ib/IIa trial focused on the novel TGF-β1 receptor kinase inhibitor, vactosertib (TEW-7197), combined with durvalumab (PD-L1 antagonist) in patients (*n* = 60) with advanced, PD-L1-positive NSCLC (NCT03732274, scheduled for completion in December 2022) (Huang et al., 2021).

Targeting of MMP-9 represents another, albeit unproven in the clinical setting, strategy to neutralize TGF-β1. In this context, MMP-9 derived from tumor cells, including NSCLC (Liu et al., 2010) and neutrophils, promotes proteolytic activation (Kobayashi et al., 2014) of latent TGF-β1 in the TME, resulting in immunosuppression and tumor progression (Juric et al., 2018; Germann et al., 2020). Despite the disappointments experienced with the earlier series of Zn^2+^-chelating MMP inhibitors, development and pre-clinical assessment of MMP-targeted mAbs and small molecule inhibitors with alternative sites of action is ongoing, raising the possibility of pending acquisition of a novel agent that targets neutrophil MMP-9 (Fields, 2019).

The recent development of bintrafusp alfa (M7824) also represented a promising innovation in the dual therapeutic targeting of TGF-β1 and PD-L1 in cancer. It is a first-in-class bifunctional fusion protein construct that consists of the extracellular domain of TGF-βRII fused with a human PD-L1-targeted human immunoglobulin G1 antibody. This dual immunotherapeutic agent appeared to hold considerable promise, demonstrating “encouraging efficacy and tolerability” in a small phase I clinical trial undertaken in patients with NSCLC who had been previously treated with platinum (Paz-Ares et al., 2020b). However, the results of a larger phase III clinical trial initiated in 2018 encompassing 584 participants proved to be extremely disappointing (NCT03631706). This trial, known as INTR@PID Lung 037, compared the therapeutic efficacy of M7824 with that of the PD-1-targeted ICI, pembrolizumab alone, in patients with stage IV NSCLC who had high level of expression of PD-L1 (NIH, 2021b). However, following the recommendation of the Independent Data Monitoring Committee, the trial was terminated on January 19, 2021 due to the improbability of M7824 meeting the major primary endpoint of PFS.

It is to be hoped that the disappointment emanating from the M7824 NSCLC clinical trial does not represent a deterrent, but rather provides the impetus to energise the development of novel, effective bi- and multi-functional therapeutic anti-cancer agents.

### Targeting Chemokine Receptors

The closely related chemokine receptors, CXCR1 and CXCR2, expressed on neutrophils and MDSCs are responsive to several chemokines produced by tumor cells and other cell types (Jaffer and Ma, 2016) and are now well recognized as viable targets in the adjunctive therapy of cancer. To our knowledge, three synthetic, small molecule, dual CXCR1/CXCR2 antagonists are undergoing extensive evaluation in the preclinical and early phase clinical trial settings. These are reparixin (Casilli et al., 2005), SX-682 (Lu et al., 2017) and navarixin (MK-7123) (Rennard et al., 2015). In the case of SX-682, this agent has been shown in murine models of cancer therapy, including NSCLC, to be most effective when used in combination with immunotherapy, specifically ICI-targeted mAbs (Kargl et al., 2019) and natural killer-based immunotherapy (Greene et al., 2020). As mentioned, all three of these pharmacological antagonists of CXCR1/2 receptors are currently being evaluated in early phase clinical trials. These are focused on different types of advanced malignancy, usually as adjuncts to immunotherapy, chemotherapy and/or radiotherapy. One of these (NCT03473925, estimated completion date May 15, 2021) is a phase II clinical trial in which navarixin in combination with the PD-1 antagonist, pembrolizumab, is being investigated in adults (*n* = 120) with PD-L1-positive, refractory NSCLC, as well as in castration-resistant prostate cancer and microsatellite stable colorectal cancer (NIH, 2021a).

Although not yet proven in the clinical setting of NSCLC, two preclinical studies have demonstrated the therapeutic efficacy of SX-682 in the adjunctive, experimental therapy of NSCLC when used in combination with immunotherapy. In the first of these (mentioned earlier) Kargl et al. (2019) compared the therapeutic efficacies of a PD-1- targeted mAb and SX-682 used individually or in combination. Only the anti-PD-1/SX-682 combination demonstrated therapeutic efficacy in the murine model of NSCLC as demonstrated by increased influx of anti-CD8^+^ T cells and a significant reduction in tumor burden. In another more recent study, Horn et al. (2020) demonstrated the therapeutic efficacy of SX-682 in combination with M7824 in murine models of both NSCLC and breast cancer.

### Targeting Methylglyoxal

Very recently Baumann et al. (2020) working on mechanisms of induction of immunological tolerance in experimental organ transplantation, a setting in which MDSCs also play an immunosuppressive role, described a novel mechanism by which these cells suppress T cell reactivity (Baumann et al., 2020). These authors observed that due to their state of relative metabolic dormancy, MDSCs produce high levels of the toxic, reactive aldehyde, methylglyoxal from glucose. Accumulation of methylglyoxal in MDSCs resulted from slow rates of glycolysis and mitochondrial respiration, inducing a state of metabolic dormancy. This state of metabolic dormancy was transferred to T cells following co-culture with MDSCs, and appeared to result from depletion of T cell cytosolic arginine. Intriguingly, addition of metformin reversed metabolic dormancy in both cell types and recovery of T cell function (Baumann et al., 2020).

With respect to neutralization of MDSCs by metformin in cancer, there is an awareness that diabetic patients treated with this agent appear to have a lesser predisposition to develop various types of cancer (Saraei et al., 2019). Although mechanisms remain to be firmly established, interference with the intracellular generation of methylglyoxal by MDSCs (Baumann et al., 2020), as well as inhibition of STAT3 (Xu et al., 2019) are possibilities. Interestingly, inhibition of STAT3 by metformin may also imply the potential of this agent to downregulate the expression of FATP2 as described by Adeshakin et al. (2020). With respect to clinical studies, we are aware of only one small phase II trial encompassing 139 diabetes-free patients with advanced epidermal growth factor receptor (EGFR)-mutated lung adenocarcinoma that investigated the therapeutic potential of EGFR tyrosine kinase inhibitors (TKIs) alone and in combination with metformin (NCT03071705) (Arrieta et al., 2019). The authors observed that addition of metformin to the TKIs, erlotinib hydrochloride, afatinib dimaleate, or gefitinib, resulted in significant prolongation of both PFS (median:13.1 vs 9.9 months, *p* = 0.03) and OS (median: 31.7 vs 17.5 months, *p* = 0.02). While conceding the limitations of their small under-powered, study, the authors propose that their findings justify more intensive investigation, specifically using an adequately powered phase III study design (Arrieta et al., 2019).

### Interleukins-1β and 17A as Neutrophil/Myeloid-Derived Suppressor Cells-Targeted Strategies

Both of these pro-inflammatory cytokines have been implicated in promoting the influx of pro-tumorigenic neutrophils/MDSCs into the TME. In the case of IL-1β, murine tumors that express this cytokine are highly infiltrated by granulocytic MDSCs (Tannenbaum et al., 2019). This is achieved by IL-1β-driven expansion of these cells via secretion of the growth factors G-CSF and Bv8, as well as CXCR2 interacting chemokines and upregulation of expression of vascular endothelial adhesion molecules. All of these mechanisms favor neutrophil recruitment and expansion of MDSCs (Tannenbaum et al., 2019).

The involvement of IL-1β in the pathogenesis of immune dysfunction in NSCLC was discovered inadvertently. This occurred following secondary analysis of data from a clinical trial focused on the therapeutic efficacy of the IL-1β-targeted mAb, canakinumab, in a large cohort (*n* = 10,061) of atherosclerosis patients who had experienced a prior myocardial infarction and were free of cancer at the outset (Ridker et al., 2017). The secondary analysis revealed that patients in the canakinumab treatment group experienced significant reductions in incident lung cancer and cancer mortality (Ridker et al., 2017). These findings resulted in initiation of the CANOPY Trial Program, which is a series of three major, international phase III clinical trials. These are focused on the therapeutic efficacy of canakinumab alone or in combination with other anti-cancer agents such as pembrolizumab and platinum-based chemotherapy in patients with advanced NSCLC (Paz-Ares et al., 2020a). Two of these trials (NCT03626545, Canopy-2) and NCT03631199, Canopy-1) are scheduled to be completed during 2022 and the third (NCT03447769, Canopy-A) in early 2027. Following an interim analysis of data, however, Novartis International AG announced on March 09, 2021 that NCT03626545 (randomized, double-blind, placebo-controlled trial encompassing 245 patients) had failed to achieve the primary end-point of OS in patients with locally advanced NSCLC. Patients in this study were treated with either the combination of canakinumab and docetaxel, or with placebo/docetaxel in the case of the control group. Although uncertain, the lack of therapeutic efficacy of canakinumab may relate to the fact this cohort of NSCLC patients had very advanced, refractory disease.

Given its key role in promoting expression of IL-8 by various cell types, including tumor cells (Inozume et al., 2009) that promote influx of neutrophils/MDSCs into the TME (He et al., 2010), IL-17A also represents a prominent target for the control of neutrophilic inflammation. With respect to NSCLC, a preclinical study by Akbay *et al.* based on a murine model of lung tumorigenesis revealed that tumor production of IL-17A and neutrophil influx were key events not only in disease progression, but also in mediating resistance to PD-1-targeted immunotherapy (Akbay et al., 2017). These findings are consistent with an earlier description of IL-17A being “a central regulator of lung tumor growth” (Reppert et al., 2012). In this context, Chen *et al.* recently reported that the numbers of circulating Th17 cells and serum levels of IL-17A were significantly higher in patients with NSCLC relative to those of healthy control subjects and correlated with disease type (squamous cell carcinoma vs adenocarcinoma) and stage (Chen et al., 2020).

Given the increasing recognition of the involvement of neutrophils in disease initiation and progression, as well as resistance to therapy, it is surprising that IL-17A does not appear to have been investigated as a drug target for lung cancer. Even more so, given the availability of mAbs that target IL-17A and its receptor and their well-recognized therapeutic efficacy in the treatment of psoriasis, a disease in which neutrophils play a key pathogenetic role (Watanabe et al., 2020).

### Antagonism of Platelet Activation

Aside from their critical role in hemostasis, platelets are now recognized as being key players in promoting inflammatory responses, acting in partnership with neutrophils to drive key pro-tumorigenic activities, particularly production of ROS and induction of NET formation (Lisman, 2018; Zucoloto and Jenne, 2019). When produced in the TME, NETs provide a protective barrier to tumor cells, preventing access of anti-tumor TILs (Demers et al., 2016; Teijeira et al., 2020). Inflammation-mediated damage to vascular endothelium, as well as intravascular trapping of tumor cells by NETs also favors metastasis (Cools-Lartigue et al., 2013).

Although a possible role for platelets in augmenting the pro-tumorigenic potential of neutrophils in NSCLC remains largely unexplored, evidence exists, albeit limited, that supports this contention (Schmied et al., 2021). In this context, (Yu et al., 2013a)*.,* in an earlier retrospective study, reported that the circulating platelet count was a predictor of prognosis in operable NSCLC (*n* = 510 patients) (Yu et al., 2013a). Patients in the sub-groups with higher platelet counts had an increased risk of disease progression (HR = 1,58; 95% CI = 1,015–2,453) (Yu et al., 2013a). More recently, pre-therapy thrombocytosis was identified as a negative indicator for survival in a cohort (*n* = 70) of patients with advanced NSCLC treated with first-line chemotherapy (Khan et al., 2020). In another study (*n* = 237 patients), an elevated platelet:lymphocyte ratio was found to be independently associated with poor survival in stage IV NSCLC associated with malignant pleural effusion (Lim et al., 2019).

While we concede that no compelling evidence currently exists linking platelet numbers and activation status to the immunosuppressive, pro-tumorigenic activity of neutrophils, it is noteworthy that the use of aspirin and anti-platelet agents for cancer prevention and treatment has been advocated by many clinical investigators (Xu et al., 2018).

### Manipulation of Fecal Microbiota as a Potential Strategy

The intriguing association between the composition and diversity of the microflora of the gut microbiome and the responsiveness of several types of advanced malignancies to innovative immunotherapies, particularly ICI-targeted mAbs, is now well-recognized (Derosa et al., 2018). In this context, three independent, seminal studies published concurrently in “Science” in January 2018 are notable (Gopalakrishnan et al., 2018; Matson et al., 2018; Routy et al., 2018). Using metagenomics, all three studies identified favorable relationships between specific types of colonic commensal bacteria with beneficial anti-tumor immune responses and clinical outcomes following initiation of PD-1/PD-L1-targeted immunotherapy in patients mostly with metastatic melanoma or urothelial cancer. These findings were replicated in studies involving transplantation of germ-free mice with feces from human cancer patients who had responded to PD-1-targeted immunotherapy (Gopalakrishnan et al., 2018; Matson et al., 2018; Routy et al., 2018).

More recently and although somewhat premature, the findings of one small very recently published report indicated that fecal transplantation may represent a possible strategy to nullify MDSCs (Davar et al., 2021). This was a pilot, single-arm clinical trial encompassing 15 patients with advanced PD-1-targeted-refractory melanoma. All patients were treated with a single dose of a pooled fecal microbiota transplant (FMT) delivered colonoscopically together with pembrolizumab, which was administered at three-weekly intervals until recovery or development of intolerable toxicity. The pooled FMT was prepared from the FMTs of patients who had previously responded to PD-1-targeted therapy. Six patients who were classified as responders were colonized by taxa (*Faecalibacterium prausnitzii; Akkermannsia muciniphila*) known to be associated with PD-1 responsiveness. Clinical improvement was associated with increased numbers and activity of CD8^+^ T cells, together with decreased circulating levels of IL-8 and decreased numbers of MDSC-like, IL-8-expressing cells in the TME (Davar et al., 2021). Non-responders remained colonized with unfavorable taxa, specifically those of the *Bacteroides* genus.

Although unexplained mechanistically and requiring stringent confirmation in NSCLC and other types of malignancy, this innovative research may have identified another novel strategy to neutralize immunosuppressive neutrophils/MDSCs. Surprisingly, however, NLR values measured prior to and during administration of FMT/pembrolizumab were not reported.

The aforementioned various putative strategies aimed at attenuating the immunosuppressive, pro-tumorigenic potential of neutrophils/MDSCs in NSCLC, which have entered either the preclinical or early phases of clinical evaluation (some recently abandoned, while others are mostly ongoing) are summarized in Tables 1 and 2, respectively.

**TABLE 1:** Potential targets and pharmacological/biological strategies to attenuate the immunosuppressive, pro-tumorigenic activities of neutrophils and MDSCs.

Target	Inhibitor	Mechanism	References
Granulocyte-macrophage colony-stimulating factor	Lipofermata	Inhibitor of FATP2, limited by toxicity concerns	Veglia et al. (2019), Adeshakin et al. (2020)
	Metformin	Inhibitor of STAT3 leading to downregulation of FATP2	
	Prostaglandin synthetase inhibitors	Inhibit generation of immunosuppressive PGE_2_ associated with upregulation of FATP2	
Transforming growth factor-β1	Vactosertib; Fresolimumab	Inhibitor of TGF-βRI kinase Neutralizes all 3 isoforms of TGF-β	Huang et al. (2021)
	MMP-9 antagonists (in pipeline)	Inhibit activation of latent TGF-β1	Kobayashi et al. (2014), Juric et al. (2018), Fields (2019), Germann et al. (2020)
Chemokine receptors CXCR1 and CXCR2	Navarixin, reparixin, SX-682	Chemokine receptor antagonists	Casilli et al. (2005), Lu et al. (2017), Rennard et al. (2015), Kargl et al. (2019), Horn et al. (2020)
Methylglyoxal	Metformin	Reverses metabolic dormancy and depletion of arginine in MDSC-exposed T cells	Baumann et al. (2020)
Interleukin-1β	Monoclonal antibodies that target IL-1β and its receptor	Antagonize infiltration of neutrophils and MDSCs into the TME	Tannenbaum et al. (2019)
Interleukin-17 A	Monoclonal antibodies that target IL-17A and its receptor	Antagonize infiltration of neutrophils and MDSCs into the TME	Inozun et al. (2009), He et al. (2010), Akbay et al. (2017), Chen et al. (2020)
Platelets	Inhibitors of platelet activation	Antagonize platelet-mediated neutrophil activation	Yu et al. (2013a), Lim et al. (2019), Khan et al. (2020), Schmied et al. (2021)
Immunosuppressive gut commensal organisms	Fecal microbiota transplantation	Re-colonize colon with organisms that favorably re-program the immune system	Gopalakrishnan et al. (2018), Matson et al. (2018), Routy et al. (2018)

FATP2, fatty acid transport protein 2; MDSC, myeloid-derived suppressor cell; MMP-9, matrix metalloproteinase-9; PGE2, prostaglandin E2; TME, tumor microenvironment.

**TABLE 2:** Summary of clinical trials in NSCLC with putative inhibitors of neutrophil/MDSC activity.

Agent	Target	Study phase	Participant numbers	NCT number	Study population	Outcome	References/Sponsor
Galunisertib (+ nivolumab)	TGF-β1 receptor kinase	II	Unclear	02,423,343	Recurrent/refractory NSCLC	Terminated prematurely due to cardiotoxicity	Eli Lilly Company announcement
							30 January 2020
Vactosertib (TEW-7197) (+ durvalumab)	TGF-β1 receptor kinase	Ib/IIa	60	03,732,274	Advanced PD-L1-positive NSCLC	Ongoing	Huang et al. (2021)
Fresolimumab (+ radiotherapy)	Antagonist of all three isoforms of TGF-β	I/II	60	02,581,787	Early stage NSCLC	Ongoing	Huang et al. (2021)
Bintrafusp alfa (also known as M7824)	Dual inhibitor of TGF-βRII and PD-L1	III	584	03,631,706	Stage IV NSCLC	Terminated prematurely due to failure to achieve primary endpoint of PFS	Merck Global announcement 13 January 2021
Navarixin (+ pembrolizumab)	Antagonist of CXCR1/CXCR2 chemokine receptors	II	120	03,773,925	PD-L1-positive, refractory NSCLC	Ongoing, due to be completed during May 2021	MSD Corp
Metformin (in combination with TKIs vs TKIs alone)	Possible antagonist of formation of MDSCs	II	139	03,071,705	Diabetes-free, EGFR-mutated lung adenocarcinoma patients	Metformin adjuvant therapy associated with significant prolongation of PS and OS	Arrieta et al. (2019)
Canakinumab (Canopy-A comparison of the efficacy and safety of canakinumab vs placebo as adjuvant therapy)	Antagonist of IL-1β	III	1500	03,447,769	Stages IIA—IIIA and IIIB completely resected NSCLC	Ongoing	Paz-Ares et al. (2020a)
Canakinumab (Canopy-1	Antagonist of IL-1β	III	673	03,631,199	First-line treatment of locally advanced or metastatic NSCLC	Ongoing	Paz-Ares et al. (2020a)
+ pembrolizumab							
+ platinum-based doublet chemotherapy with or without canakinumab)							
Canakinumab (Canopy-2 + docetaxel)	Antagonist of IL-1β	III	245	03,626,545	Locally advanced, previously treated NSCLC as a second-or third-line therapy	Seemingly terminated due to primary endpoint of OS not being achieved	Novartis-international AG announcement
							March 09, 2021
Fecal microbiota transplantation (administered once colonoscopically combined with PD-1-targeted immunotherapy)	Promotes transition to an immunologically favorable gut microbiota	Single-arm pilot study	15	--	Advanced PD-1-targeted-refractory melanoma (not yet evaluated in NSCLC)	Colonic re-colonization associated with reponsiveness to PD-1-targeted therapy	Davar et al. (2021)

EGFR, epidermal growth factor receptor; IL-1β, Interleukin-1β; MDSC, myeloid-derived suppressor cell; OS, overall survival; PD, programmed cell death; PFS, progression-free survival; TGF-β, transforming growth factor beta; TKI, tyrosine kinase inhibitor.

## Conclusion

Non-small cell lung cancer appears to be particularly adept at recruiting neutrophils to the TME and reprogramming these cells under the influence of various factors such as GM-CSF, TGF-β1, IL-1β and IL-17A to acquire an immunosuppressive phenotype (or phenotypes) known as MDSCs of granulocytic origin and N2-type neutrophils. These cells, in turn, protect tumor cells not only by suppressing the anti-tumor activities of TILs, but also by obstructing the access of these cells to tumors. In addition, they also interfere with the anti-tumor efficacy of various types of therapy, including chemotherapy and radiation therapy, as well as anti-angiogenic therapy and immunotherapy in particular. Consequently, neutrophils are now well recognized as being viable targets in the adjunctive therapy of various types of advanced malignancy. Although promising approaches have been identified, these currently remain in the pre-clinical and early and later clinical phases of evaluation. It is hoped that the completion of these ongoing early and, more importantly, late phase clinical trials will provide clear and convincing leads. At best, however, they are likely to provide the basis for the development of well-designed and stringently structured clinical trial development programs that may accurately identify pharmacological/biological strategies that effectively target neutrophils, either directly or indirectly, in patients with NSCLC and other types of intransigent malignancy. These programs will, however, be confronted by major challenges. These include the potential of tumor infiltrating suppressor cells, including neutrophils, to transition into a range of phenotypes in the TME, most of which are currently poorly understood and have variable immunosuppressive capabilities.

Identifying novel neutrophil-associated predictive biomarkers and harmonizing these with effective neutrophil/MDSC-targeted therapies, individually or in combination, would represent a priority achievement that may contribute significantly to improving the outcome of NSCLC.
